# S1 Guideline: Therapy of generalized pustular psoriasis

**DOI:** 10.1111/ddg.70244x

**Published:** 2026-06-14

**Authors:** Rotraut Mößner, Neda Cramer, Sascha Gerdes, Ulrike Hüffmeier, Andreas Körber, Kai Krause, Christian Kromer, Katharina Antonia Langen, Sandra Philipp, Knut Schäkel, Michael P. Schön, Michael Schultheis, Lukas Sollfrank, Petra Staubach, Michael Sticherling, Johannes Wohlrab, Dagmar Wilsmann‐Theis, Cornelia Paul

**Affiliations:** ^1^ Department of Dermatology, Venereology and Allergology University Medical Center Göttingen Göttingen Germany; ^2^ Center for Inflammatory Skin Diseases, Department of Dermatology Venereology and Allergology, University Medical Center Schleswig‐Holstein, Campus Kiel Kiel Germany; ^3^ Institute of Human Genetics, Centre for Personalized Medicine and Research (CESAR), University Hospital Erlangen, Friedrich‐Alexander University Erlangen‐Nürnberg Erlangen Germany; ^4^ Hautärzte RÜ 143 Essen Germany; ^5^ Department of Dermatology and Venereology Martin Luther University Halle‐Wittenberg Halle Germany; ^6^ Dermatology Office Dres. med. M. Friedrich/S. Philipp Oranienburg Germany; ^7^ Department of Dermatology University Hospital Heidelberg Heidelberg Germany; ^8^ Department of Dermatology University Medical Center Mainz, Johannes Gutenberg University Mainz Germany; ^9^ Department of Dermatology Deutsches Zentrum Immuntherapie (DZI), University Hospital Erlangen Erlangen Germany; ^10^ Center for Skin Diseases, Department of Dermatology and Allergology University Hospital Bonn Bonn Germany; ^11^ Institute of Clinical Chemistry and Clinical Pharmacology, University Hospital Bonn Bonn Germany

**Keywords:** Genetics, GPP, guideline, therapy

## Abstract

The S1 guideline “Therapy of generalized pustular psoriasis (GPP)” is a German guideline developed in accordance with the criteria of the AWMF. The full version addresses clinical presentation, pathogenesis, diagnosis and differential diagnosis, comorbidities, and therapy. In addition, therapy recommendations for adults with GPP are provided. Both approved and off‐label therapies are considered. The present article is a shortened version of the guideline, whose  therapy recommendations are presented in full and without omission.

## Introduction

In the present guideline, general aspects of generalized pustular psoriasis, local therapy, physical treatment, and pharmacological therapy of adults are addressed. In Figure 1, the recommendations for treatment of generalized pustular psoriasis are presented in simplified form.

**FIGURE 1:**
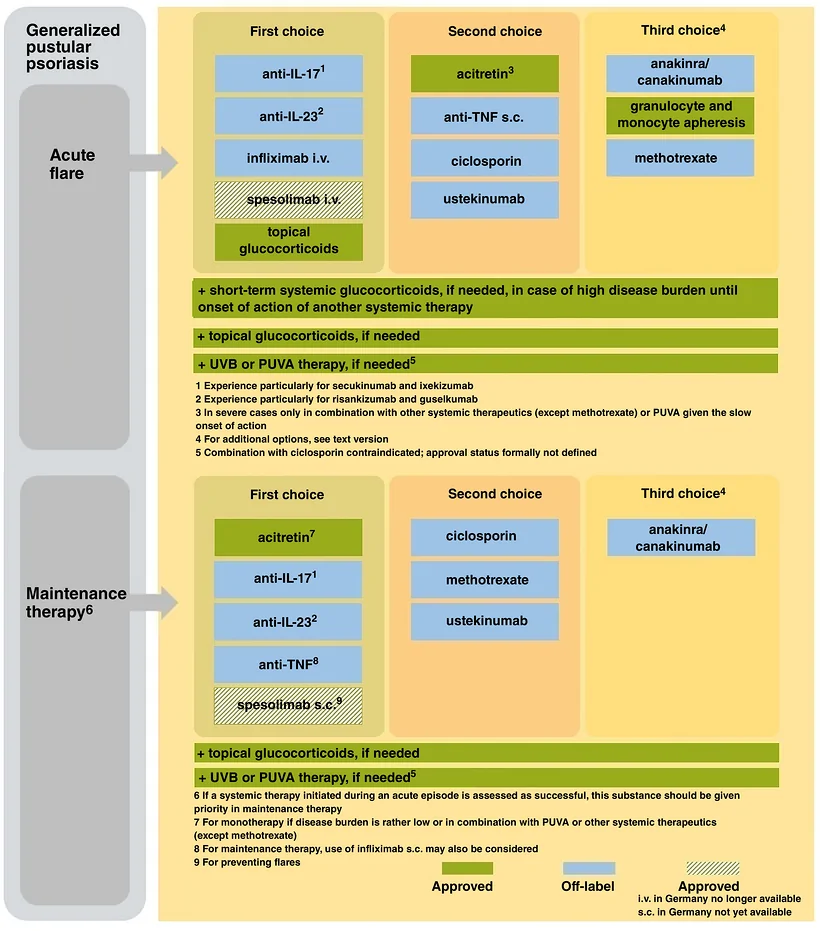
Simplified recommendations for the treatment of generalized pustular psoriasis.

The treatment recommendations are presented in full and without omission, while the content concerning medical background and available studies is presented in shortened form. The complete long version of the guideline is available on the AWMF website at https://register.awmf.org/de/leitlinien/detail/013‐107.

## Definition

Generalized pustular psoriasis (GPP) is a rare, severe, and potentially life‐threatening auto‐inflammatory neutrophilic dermatosis. It was first described by von Zumbusch as type of pustular psoriasis in 1909.^1^

The *European Rare and Severe Psoriasis Expert Network* (ERASPEN) defines GPP as widespread cutaneous erythema with sterile, macroscopically visible pustules that can be either persistent (> 3 months) or relapsing with at least two episodes. GPP may manifest with or without systemic inflammation and decreased general condition, as well as with or without psoriasis vulgaris.^2^, ^3^, ^4^, ^5^

An acute flare is understood as significant aggravation of signs and symptoms, often associated with symptoms like pain and/or decreased general condition.

## Epidemiology

Only few data on the epidemiology of GPP are available. The prevalence seems to vary depending on ethnic origin and appears to be significantly higher in Asian ethnicities compared to the European population. Based on data from 1996, the prevalence was 7.46 per million population in Japan,^6^ while a prevalence of 1.76 per million population was recorded in a French survey from 2004 including 121 dermatologic hospitals.^7^ The evaluation of the South Korean National Health Insurance database from 2011 to 2015 demonstrated a significantly higher prevalence of 88 to 124 cases per million population.^8^ No absolute numbers are available for Germany. In a review on GPP, a prevalence of 23.7 per million population was estimated based on GPP cases requiring inpatient treatment in Germany in 2019 (395 hospitalizations) and the assumption that patients with GPP experience a flare every 5 years.^9^ Given that GPP is a very rare disease with high diagnostic uncertainty, these numbers must be viewed critically. Probably, they do not correspond to epidemiologic reality. It is estimated that approximately 80 % of all psoriasis cases are attributable to psoriasis vulgaris, 20 % to pustular forms, but only 2 % to GPP with a higher prevalence in women.^5^, ^10^, ^11^, ^12^ In most cases, onset of disease is between the 40th and 60th year of life,^11^ although manifestation in childhood has also been described.^13^

## Pathogenesis

GPP is caused by an autoinflammatory reaction with deficient control of the interleukin (IL)‐36 signaling pathway and other mediators.^3^, ^9^ In many cases, infections trigger the non‐specific immune defense and initial IL‐36 production. The current model of the immunopathogenesis is summarized in Figure 2 and described in the Figure legend.

**FIGURE 2:**
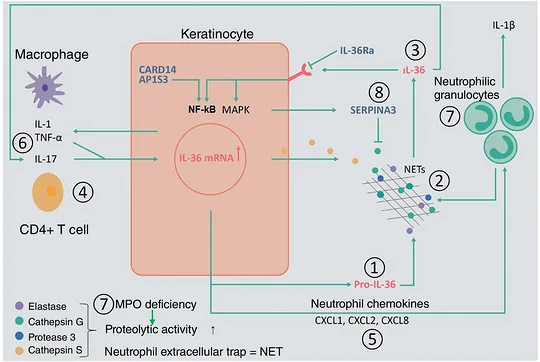
Pathogenesis of GPP (adapted from 9). IL‐36 is released by keratinocytes as an inactive precursor protein (α, β, or γ) (pro‐IL‐36) ①. It becomes biologically active after extracellular proteolytic cleavage ②.^57^ Among the IL‐36–activating proteases is cathepsin S, which is mainly produced by mononuclear phagocytes (macrophages and dendritic cells). Protease 3, cathepsin G, and elastase are additional IL‐36–activating proteases that are released particularly during inflammatory reactions by neutrophilic granulocytes, together with neutrophil extracellular traps (NETs). IL‐36 stimulates the proinflammatory activation of keratinocytes in an autocrine manner ③ and furthermore induces activation of endothelial cells, fibroblasts, and immune cells ④. The typical clinical manifestation is an eruptive appearance of pustules, which is driven by the expression of chemokines recruiting neutrophilic granulocytes ⑤. In addition, T cells and macrophages are involved in the inflammatory process ④. Together, these cells shape the local cytokine milieu with expression of IL‐36, IL‐1, TNF‐α, and IL‐17A ⑥.^57^ Myeloperoxidase (MPO) deficiency correlates with the activity of serine proteases ⑦ and leads, among other effects, to reduced NET formation ②. SERPINA3 inhibits the serine protease cathepsin G ⑧. Proteins encoded by genes of which rare variants predispose to pustular forms of psoriasis are shown in blue. *Abbr*.: IL‐36Ra, IL‐36 receptor antagonist; IL‐1β, interleukin‐1β; TNF‐α, tumor necrosis factor alpha; IL, interleukin; CARD14, caspase recruitment domain‐containing protein 14; AP‐1, adaptor protein complex 1; AP1S3, adaptor protein complex 1 subunit sigma 3; SERPINA3, serine protease inhibitor A3; NF‐κB, nuclear factor kappa‐light‐chain‐enhancer of activated B cells; MPO, myeloperoxidase; MAPK, mitogen‐activated protein kinase; CD4+, cluster of differentiation 4; NET, neutrophil extracellular trap; CXCL, C‐X‐C motif chemokine ligand.

## Genetic basics

See long version of the guideline.

## Trigger factors

Trigger factors of generalized pustular psoriasis are presented in Table 1.

**TABLE 1:** Trigger factors of generalized pustular psoriasis.

Drugs Beta blockers, antimalarial drugs, lithium, contraceptives, terbinafine, glucocorticoids
Infections
Biologics (especially TNF inhibitors, rarely interleukin‐17 or interleukin‐23 inhibitors (a so‐called paradoxical reaction))
High UV exposure
Pregnancy

## Clinical presentation and course

GPP is characterized by disseminated erythemas and swelling as well as coalescent sterile pustules (Figure 3).^1^ Often, the general condition is severely impaired and arthritis may occur. Common laboratory findings are leukocytosis with neutrophilia and significantly elevated levels of CRP and ESR, and, less commonly, hypocalcemia and hypoproteinemia.^11^, ^14^, ^15^, ^16^ In GPP, disease onset in childhood (early onset) is differentiated from onset in adulthood (adult onset).^11^, ^17^, ^18^, ^19^, ^20^

**FIGURE 3:**
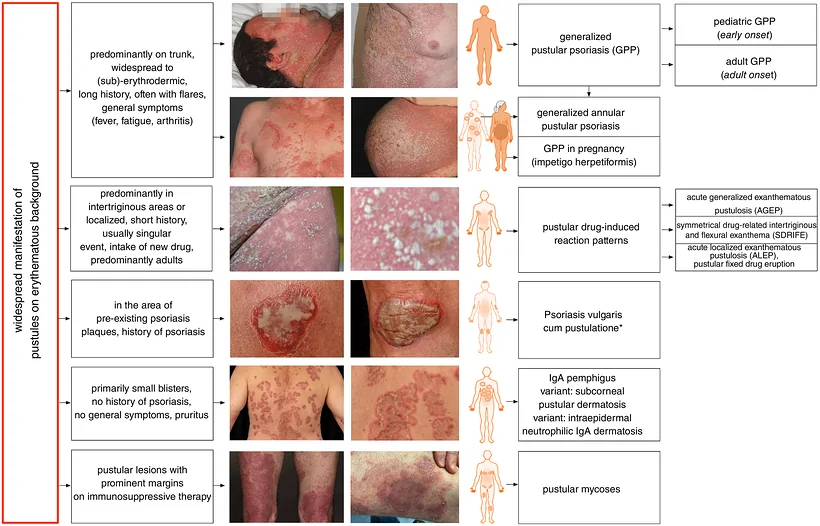
Differential diagnostic criteria. *Source: Clinical images provided by Prof. Dr. Ulrich Mrowietz.

Histologic characteristics of GPP are conspicuous accumulations of neutrophilic granulocytes in stratum spinosum (Kogoj's spongiform pustules) and stratum corneum (Munro's microabscesses). In addition, other features, such as parakeratosis, acanthosis, hyperkeratosis, elongated rete ridges, diminished stratum granulosum, and dilated capillaries in papillary dermis, which are also typical features of psoriasis vulgaris, may be detected in some cases, but may (still) be lacking in acute courses or early stages.^21^

## Comorbidity

Comorbidity in GPP is significantly affected by the burden of systemic inflammation consisting of both disease activity (severity) and disease duration (frequency and duration of flares). It includes cardiovascular diseases, such as arterial hypertension, ischemic heart disease, stroke, and metabolic diseases such as hyperlipidemia and diabetes mellitus.^17^, ^18^, ^19^ Given the substantial impairment of the quality of life in association with neuroinflammatory processes, a particular psychological burden (anxiety disorder, depression) must be assumed as well.^14^, ^22^, ^23^, ^24^, ^25^

In addition, geographic tongue as non‐specific sign of chronic inflammation is quite common.^26^, ^27^ While 17 % of the patients with GPP had psoriatic arthritis in a retrospective study from Germany, prevalences of up to approximately one third of patients have been described.^28^ GPP is currently considered to be a systemic inflammatory multisystem disease with, among other comorbidities, joint involvement.^29^ Approximately 50 % to 60 % of the patients with GPP have or had also psoriasis vulgaris.^2^, ^11^, ^30^ However, the proportion of patients with concomitant moderate to severe psoriasis vulgaris at the time of a GPP flare seems to be significantly smaller. Furthermore, coincidence with other autoimmune or autoinflammatory diseases, especially Crohn's disease and neutrophilic cholangitis, is common.^30^, ^31^, ^32^, ^33^ Histologically, neutrophilic cholangitis is defined by infiltration of the bile duct system by neutrophilic granulocytes resulting in cholestasis with icterus and increase of typical parameters of cholestasis (bilirubin, bile acids, cholesterol, bile enzymes) and cholestatic pruritus.^31^, ^34^, ^35^, ^36^, ^37^

## Complications

Epidemiologic analyses show an increased mortality in patients with GPP, and deaths attributable to GPP flares have been described in approximately 5 % to 10 % of GPP cases.^4^, ^7^, ^14^, ^15^, ^38^, ^39^, ^40^ Sepsis, rare aseptic forms of pneumonitis (acute respiratory distress syndrome), and eye inflammation (conjunctivitis, uveitis, or iritis) have been described as severe complications.^14^, ^38^, ^41^, ^42^, ^43^, ^44^, ^45^, ^46^ In severe erythrodermic courses, the risk of infection, particuclarly by bacteria, is increased. Moreover, hypoalbuminemia and edemas may occur due to loss of albumin and insufficient hepatic synthesis.^47^ In addition, alterations of calcium metabolism in the form of primary hypoparathyroidism have been described in individual cases, although it is unclear whether this represents a trigger factor or a consequence of GPP.^48^, ^49^, ^50^, ^51^

## Clinical assessment criteria (scores)

Analogous to the assessment criteria of psoriasis vulgaris, the severity of GPP is measured by *Generalized Pustular Psoriasis Area and Severity Index* (GPPASI) and *Generalized Pustular Psoriasis Physician Global Assessment* (GPPGA).^52^, ^53^ The GPPASI corresponds to the PASI with total scores from 0 to 72 with the criterion “induration” being substituted by “pustules”. The GPPGA assesses the severity of GPP using a *Physician Global Assessment* (PGA). Pustules, erythema, and scaling are assessed, and each component can achieve a score from 0 to 4.^9^

The JDASI (*Japanese Dermatological Association Severity Index*) includes systemic parameters in addition to skin lesions. A maximum of 9 points are attributed to the skin lesions (erythema area overall, erythema area with pustules, edema area) and 8 points to the systemic involvement (pyrexia, leukocyte count, CRP, serum albumin). Accordingly, a score between 0 and 17 points results (severity: 0 to 6 points: mild, 7 to 10 points: moderate, 11 to 17 points: severe).^47^

## Diagnostic evaluation

Recommendations for diagnostic evaluation are presented in Table 2.

**TABLE 2:** Recommendations for diagnostic evaluation.

**Recommendation**
Determination of potential trigger factors is recommended.
For evaluating the overall condition of patients, laboratory tests (blood count including white blood cell differential, kidney function tests, liver function tests, serum electrolytes, and CRP) are recommended.
Determination of albumin, calcium, and procalcitonin in serum may be recommended in case of decreased general condition.
Histological examination of a skin biopsy is recommended. PAS staining should be performed to exclude mycosis.
For assessing the severity, use of validated instruments (GPPASI, GPPGA, JDASI) may be recommended.
For the evaluation of AGEP as differential diagnosis, the criteria of the EuroSCAR study group are recommended.
Screening for comorbidity (cardiovascular, metabolic, psychiatric, and chronic inflammatory diseases) may be recommended.

*Abbr*.: AGEP, acute generalized exanthematous pustulosis; CRP, *C‐reactive protein*; GPPGA, *Generalized Pustular Psoriasis Physician Global Assessment*; GPPASI, *Generalized Pustular Psoriasis Area and Severity Index*; JDASI, *Japanese Dermatological Association Severity Index*; PAS *Periodic acid‐Schiff*

## Differential diagnoses

Criteria for differential diagnosis are presented in Figure 3. See also long version of the guideline

## State of medical care

See long version of the guideline.

## Therapy

### Initiation of therapy

Various modalities are used for the therapy of GPP, and some of these may be used in combination. They include topical therapies, UV therapies, medical systemic therapies, and granulocyte and monocyte adsorptive apheresis (GMA), which is barely used in Europe. While topical therapy and UV therapy may be sufficient for less pronounced forms, additional systemic therapies are required for more severe forms or recalcitrant courses. If a medical systemic therapy initiated during an acute episode is assessed as successful, this substance should be given priority in maintenance therapy. This does not apply to systemic glucocorticoids. Spesolimab should be administered according to the respective approval (i.v. versus s.c.). For combination therapies, interactions and contraindications must be considered. Specific aspects of therapy during pregnancy are addressed in a separate chapter.

Primarily, the evidence of the therapies in GPP is presented. Concerning the rapidity of onset of action, available study data and the authors’ own experience with GPP are considered. Moreover, the experience gained from other indications, such as psoriasis vulgaris, is taken into account. Concerning application instructions, laboratory tests, drug interactions, contraindications, and special aspects during treatment, we refer to the summaries of product characteristics and the guidelines on other indications. Practical aspects on the use of therapies approved for psoriasis vulgaris are addressed in detail in the current S3 guideline “Therapy of psoriasis”.^54^, ^55^ We recommend following the dosages used for psoriasis provided the drug is approved for this indication and unless specified otherwise below. So far, no German guideline or stratified treatment recommendation on GPP existed. In 2018, a guideline on GPP was published in Japan that included primarily Asian patients in the underlying data analysis.^27^ Some patients with GPP also suffer from psoriasis vulgaris or psoriatic arthritis. This should be considered when choosing the systemic therapy.

**Table:** 

**Statement** Based on the assessment of the authors of this guideline, GPP is a rare, severe, and potentially lethal disease severely impairing the quality of life of those affected. In the course of the challenging treatment, it is often necessary to use therapies that are not approved for GPP (*off‐label use*) and for which, often, no controlled studies are available. If respective comorbidities, especially psoriasis vulgaris or psoriatic arthritis, are present, the therapeutics discussed in this guideline may be used, in part, in connection with the approval for these diseases.

## Topical therapy

### Recommendation 4.2.1‐4.2.3 of the AWMF guideline

**Table:** 

For the acute GPP flare, we recommend glucocorticoids of class IV (clobetasol). Given their unfavorable therapeutic index, they should be used only short‐term for treating an acute flare, if possible, especially until onset of action of systemic therapies. For milder flares and medium‐ or long‐term treatment we recommend topical glucocorticoids of class II or III with high therapeutic index, such as prednicarbate or mometasone furoate.^56^ Long‐term extensive application of glucocorticoids, especially as monotherapy, should be avoided, if possible.
Topical therapy with vitamin D3 analogs (*off‐label*) in acute GPP is not recommended. Their use, also as fixed‐dose combination with a glucocorticoid (*off‐label*), may be considered as maintenance therapy and in cases with lower disease burden of GPP.
Topical tacrolimus (*off‐label*) may be considered in individual cases for medium‐ or long‐term therapy of GPP, for example, in patients with steroid phobia or if sensitive areas, such as face and genitals, are affected.

For background information, we refer to the long version of the guideline.

## Physical therapy

### Recommendation 4.3.1.‐4.3.2 of the AWMF guideline

**Table:** 

Psoralen and UVA (PUVA) therapy may be considered in GPP primarily for stabilizing a treatment success given the rather slow onset of action. It may be combined with other systemic therapies, with the use in combination with ciclosporin being contraindicated. With respect to side effect profile and practicability for the patient, PUVA bath therapy may be advantageous compared to oral PUVA.
UVB phototherapy may be recommended especially for stabilizing a treatment success, as combination therapy if patients respond insufficiently to systemic therapy, or if systemic therapy is not wanted by the patient.
Granulocyte and monocyte adsorptive apheresis (GMA) may be recommended if systemic therapies are insufficiently effective or contraindicated.

For background information, we refer to the long version of the guideline.

## Systemic therapy

For background information and a compilation of prospective clinical studies, we refer to the long version of the guideline.

## Systemic glucocorticoids

### Recommendation 4.4.1.1 of the AWMF guideline

**Table:** 

Cautious use of systemic glucocorticoids in GPP is recommended. They may be administered short‐term in an acute flare, especially for bridging the gap until onset of action of other therapies. If possible, they should be used in combination with other systemic therapeutics or UV phototherapy in order to also reduce the probability of flares after discontinuation. Based on the experience of the authors, however, topical treatment with glucocorticoids of class IV may be sufficient for short‐term bridging in some patients. Use of systemic glucocorticoids for maintenance therapy in GPP is not recommended.

## Retinoids

### Recommendation 4.4.1.2 of the AWMF guideline

**Table:** 

Acitretin is recommended for maintenance therapy in patients with GPP. For therapy of flares, acitretin may be recommended as second‐line therapy, given that drugs with a more rapid onset of action should be preferred in severe acute forms. Because of the teratogenicity of retinoids, other therapies should be preferred in women of childbearing age.
Etretinate is not recommended for therapy of GPP, given that acitretin has been generally accepted as alternative retinoid with shorter half‐life in Germany. Etretinate is no longer commercially available in Germany.
Isotretinoin may be considered as third‐line therapy of GPP, provided that acitretin cannot be used for substance‐specific reasons, for example, the long‐lasting teratogenic effect after discontinuation.

## Ciclosporin

### Recommendation 4.4.1.3 of the AWMF guideline

**Table:** 

Therapy with ciclosporin (CsA) may be recommended as second choice in an acute flare and temporarily also as second choice for maintenance therapy.

## Methotrexate

### Recommendation 4.4.1.4 of the AWMF guideline

**Table:** 

Methotrexate (MTX) is effective in GPP with a known safety profile. It may be recommended as second‐line treatment for maintenance therapy. Administration of MTX may be considered for therapy of a GPP flare (*off‐label*).

## Apremilast, dapsone and mycophenolate mofetil

### Recommendation 4.4.1.7 of the AWMF guideline

**Table:** 

The phosphodiesterase 4 (PDE4) inhibitor apremilast (*off‐label*) or dapsone may be considered as third‐line therapeutics in GPP. Mycophenolate mofetil (*off‐label*) is not recommended.

## Biologics

*Tumor necrosis factor (TNF) inhibitors*

### Recommendation 4.4.2.1 of the AWMF guideline

**Table:** 

For the acute GPP flare, the TNF inhibitor adalimumab (*off‐label*) may be recommended as second‐line therapeutic due to its slower onset of action compared to infliximab i.v. For maintenance therapy, adalimumab (*off‐label*) is recommended as first‐line therapy. If the effectiveness is insufficient, the dose may be increased to 80 mg every 2 weeks or 40 mg per week s.c.
The TNF inhibitor certolizumab (*off‐label*) is recommended as first‐line treatment for maintenance therapy in GPP. If the effectiveness is insufficient, the dose of 200 mg may be increased to 400 mg every 2 weeks. For the acute GPP flare, certolizumab (*off‐label*) may be recommended as second‐line therapeutic due to its slower onset of action compared to infliximab i.v. This medication plays an important role in pregnant patients.
The TNF inhibitor etanercept (*off‐label*) is recommended as first‐line medication for maintenance therapy in GPP. For the acute flare, it may be recommended as second‐line therapy (*off‐label*).
The TNF‐Inhibitor infliximab (*off‐label*) is recommended as first‐line therapy for both treatment of the acute GPP flare and maintenance therapy. Treatment of GPP flares with infliximab i.v. is recommended, given the rapid response and the weight‐adapted dosage. In case of a stable, good outcome, switching an initiated intravenous administration of infliximab to subcutaneous administration may be recommended.

## Ustekinumab

### Recommendation 4.4.2.2 of the AWMF guideline

**Table:** 

The IL‐12 and IL‐23 antagonist ustekinumab *(off‐label*) may be recommended as second‐line therapy for the acute flare and as maintenance therapy of GPP.

## IL‐17 and IL‐17R antagonists

### Recommendation 4.4.2.3 of the AWMF guideline

**Table:** 

The IL‐17A antagonists secukinumab (*off‐label*) and ixekizumab (*off‐label*) are recommended for both treatment of a GPP flare and maintenance therapy. The IL‐17 receptor antagonist brodalumab may be recommended for both treatment of a GPP flare and maintenance therapy. The IL‐17A and IL‐17F antagonist bimekizumab may be recommended for both treatment of a GPP flare and maintenance therapy of GPP.

## IL‐23 antagonists

### Recommendation 4.4.2.4 of the AWMF guideline

**Table:** 

The IL‐23 antagonists risankizumab (*off‐label*) and guselkumab (*off‐label*) are recommended for both treatment of GPP flares and maintenance therapy. The IL‐23 antagonist tildrakizumab (*off‐label*) may be considered for both treatment of GPP flares and maintenance therapy.

## IL‐1 inhibitors

### Recommendation 4.4.2.5 of the AWMF guideline

**Table:** 

Therapy of GPP with the IL‐1 receptor antagonist anakinra (*off‐label*) or the anti‐IL‐1β antibody canakinumab (*off‐label*) may be considered as third‐line therapy, that is, provided that first‐ or second‐line therapies were not successful, cannot be conducted, or if no adequate response is anticipated. Therapy with the IL‐1β inhibitor gevokizumab is not recommended, given that it is not approved by the *European Medicines Agency* (EMA) and no benefit is apparent compared to the biologics anakinra and canakinumab approved for other indications.

## IL‐36 receptor antagonists

### Recommendation 4.4.2.6 of the AWMF guideline

**Table:** 

Given its approval for this indication, the anti‐IL‐36 receptor antibody spesolimab i.v. is recommended as first‐line therapy for GPP flares. For additional flares, repeat administration of spesolimab i.v. is recommended. If there is a therapeutic response to i.v. administration(s), switching to spesolimab s.c. for maintenance therapy is recommended. Given its approval for this indication, spesolimab s.c. is recommended as first‐line therapy for preventing GPP flares, implying that use of spesolimab s.c. after GPP flares is recommended if clinical activity of GPP is then absent or low. After withdrawal of spesolimab i.v. from the market in Germany, spesolimab i.v. can only be obtained through international pharmacies. Assumption of costs should be clarified with the health insurer in advance. For spesolimab s.c., we recommend a similar approach in casethere should be no market availability in Germany in the future.

## Therapy during pregnancy

### Recommendation 4.5 of the AWMF guideline

For severe courses during pregnancy, we recommend certolizumab s.c. as first‐line treatment. Administration of adalimumab or infliximab may be recommended as second‐line therapy. Therapy with ustekinumab may be considered.

While prednisolone may be considered as complementary treatment for bridging particularly in severe courses, it is not suitable for long‐term treatment. In general, therapy with CsA may also be considered. However, CsA has a worse safety profile than the mentioned alternatives. Acitretin and MTX are absolutely contraindicated during pregnancy and their use is not recommended. For mild courses, we recommend topical application of glucocorticoids. Similarly, UVB therapy may be recommended for less pronounced skin findings or bridging. In severe courses, this therapy may be recommended in addition to biologics. Close interdisciplinary medical monitoring of the pregnant woman is recommended.

For background information, we refer to the long version of the guideline.

## CONFLICT OF INTEREST STATEMENT

A table listing the conflicts of interest of all involved parties is available in the long version of the guideline.
